# Food allergy and anaphylaxis – 2038. Insect and sting allergy

**DOI:** 10.1186/1939-4551-6-S1-P123

**Published:** 2013-04-23

**Authors:** Syed Arham Husain

**Affiliations:** 1 Aaram Ent Clinic, Bhopal, India

## Background

Insect and sting allergy in immunocompetent populations. A comparison.

## Methods

Observation made in diferent subsets of population who had insect and sting allergy (bees, wasps, scorpions, mosquitos parasites etc).

1) Immunocompetent population in the developing world engaged in farming and related activities regularly exposed to all gradient of allergens like dust molds, dander, pets, etc and when they are exposed to insect and mosquito bites, bees and wasps stings even scorpion bites they suffer to a lesser extent.

2) Immunocompromised population in the developed world far away from natural gradient of exposure of nearly all the allergens and when they are exposed to scorpion bite or bees and wasp stings there are reports of fatal reactions and even mosquito and insect bites causes serious wheal and flare reaction, this observation was made specially on the second generation of children from parents of Indian origin now living in USA and other affluent countries, where there is minimal exposure to all the allergens.

## Results

Based on the observation made on nearly 2 lakh patients it was observed that there is a direct relationship of levels of exposure to all the natural allergens and the immunotolerance achieved by this natural exposure or natural immunotherapy.

## Conclusions

After above mentioned observations we reached to the conclusion that exposure to natural gradient of allergens, insect bites mosquito bites is troublesome for the immunocompromised populations who follow avoidance of exposure to allergens as a rule and this is a disaster for them there are many deaths reported due to the above mantioned exposure, at the same time the immunotolerant population which is exposed to all sorts of allergens insect bites mosquito bites bees and wasps stings suffers to a lesser extent.

